# Wellness Forecasting by External and Internal Workloads in Elite Soccer Players: A Machine Learning Approach

**DOI:** 10.3389/fphys.2022.896928

**Published:** 2022-06-15

**Authors:** Alessio Rossi, Enrico Perri, Luca Pappalardo, Paolo Cintia, Giampietro Alberti, Darcy Norman, F. Marcello Iaia

**Affiliations:** ^1^ Department of Computer Science, University of Pisa, Pisa, Italy; ^2^ Department of Biomedical Science for Health, Università degli Studi di Milano, Milano, Italy; ^3^ Institute of Information Science and Technologies (ISTI), National Research Council of Italy (CNR), Pisa, Italy; ^4^ United States Soccer Federation, Chicago, IL, United States; ^5^ Kitman Labs, Dublin, Ireland

**Keywords:** external workload, recovery, prediction, training load, wellness

## Abstract

Training for success has increasingly become a balance between maintaining high performance standards and avoiding the negative consequences of accumulated fatigue. The aim of this study is to develop a big data analytics framework to predict players’ wellness according to the external and internal workloads performed in previous days. Such a framework is useful for coaches and staff to simulate the players’ response to scheduled training in order to adapt the training stimulus to the players’ fatigue response. 17 players competing in the Italian championship (Serie A) were recruited for this study. Players’ Global Position System (GPS) data was recorded during each training and match. Moreover, every morning each player has filled in a questionnaire about their perceived wellness (WI) that consists of a 7-point Likert scale for 4 items (fatigue, sleep, stress, and muscle soreness). Finally, the rate of perceived exertion (RPE) was used to assess the effort performed by the players after each training or match. The main findings of this study are that it is possible to accurately estimate players’ WI considering their workload history as input. The machine learning framework proposed in this study is useful for sports scientists, athletic trainers, and coaches to maximise the periodization of the training based on the physiological requests of a specific period of the season.

## 1 Introduction

Modern soccer, particularly at the elite level, is ferociously competitive. Training for success has increasingly become a balance between maintaining high performance standards and avoiding the negative consequences of accumulated fatigue (Kellmann et al., 2018; Garcia et al., 2022). Consequently, focus is increasingly being given to the monitoring of responses to training and competition load, assessment of fatigue and recovery status of athletes. Monitoring athletes’ early-signs of fatigue is important to training scheduling and can help to balance training and recovery periods. Various approaches have been proposed within sport research, including biochemical, hormonal, neuromuscular, cardiovascular, and psychosocial monitoring to prevent the undesired negative outcome of hard physical load (McLean et al., 2010; Govus et al., 2017; McKay et al., 2021). However, only few monitoring tools are reported to be sensitive to the variability of training load (Clemente et al., 2019; Op De Beéck et al., 2019; Clemente et al., 2020; Perri et al., 2021).

Nowadays, subjective measures for monitoring the athletes’ wellness and recovery status are widely used by sports practitioners due to the fact that they are cheap and simple to implement with respect to salivary, blood, or performance tests. Even though the subjective characteristics of such measures, they still permit an accurate estimate of the objective athletes’ recovery and wellness (Saw et al., 2016). For this reason, wellness questionnaires are more and more used to evaluate the physiological stress associated with physical activity in soccer by assessing their muscle soreness, sleep, fatigue, mood, energy, and more. Gallo et al. (2017) found that high intensity physical activity such as a soccer match required up to 4 days to recover. In particular, they reported that the wellness status measured before the training session affected the individual average speed during the daily session. Similarly, Perri et al. (2021) reported that the wellness index (overall wellbeing perception determined by summing the 5-point Likert scale of different areas: fatigue, sleep quality, muscle soreness, stress levels, mood) is affected by the training load (rate of perceived exertion x training duration) performed the days before. Additionally, Nobari et al. (2020), Fernandes et al. (2021), Nobari et al. (2021), and Nobari et al. (2022) showed that high training intensity negatively affects the wellbeing status of male adult semi-professional, female elite, and male young soccer players. Moreover, several items usually assessed in the wellness questionnaires, e.g., sleep quality, fatigue, and social stress, were reported to be predictive to reduction of performance in the following weeks (Coutts and Reaburn, 2008). As a matter of fact, the reduction of subjective wellness status are found to be associated with decreased countermovement jump (CMJ) performance, alterations in redox homeostasis, cortisol, creatine kinase and leukocytes (Saw et al., 2016; McKay et al., 2021). These results corroborating the fact that the wellness and recovery status affect the athletes’ performance and were affected by the training load (TL) performed in previous days.

Thanks to the technological advent of the last decades, we are now able to passively obtain a huge quantity of objective information about the external workloads during training and matches by using the global position system (GPS). Because players’ health status is affected by several factors linked to the complex human responses to external stimuli, the possibility to have this huge quantity of information might permit a complete overview of their status, which was not possible by using the single training workload feature (Duignan et al., 2020). The major limit of the previous studies that aim to detect the relationship between training workloads and players’ wellness status is that their analytical approach are mono-dimensional, i.e., they use just one variable at time without fully exploiting the complex patterns underlying the available data. Hence, the simplification hides the complexity of the training stimuli, not allowing detecting complex patterns in training workloads. However, with more information now available from sports-related research and technologies, exercise scientists and coaches have an increasing amount of data available that can be difficult to translate into useful information. For this reason, the literature about data mining and machine learning approaches is quickly growing since the last decade. These approaches could help to have a complete overview of players’ wellness, permitting the development of mathematical models able to provide accurate prediction and consequently useful insights about injury risks (Rossi et al., 2018; Ayala et al., 2019; Pappalardo et al., 2019; Seow et al., 2020; Vallance et al., 2020; Van Eetvelde et al., 2021; Rossi et al., 2022) and internal training load (Rossi et al., 2016; Rossi et al., 2017; Rossi et al., 2019).

Based on the results found on previous studies highlighting the strong mono-dimensional relationship between training workloads and individuals’ wellbeing, the aim of this study is to develop a framework of big data analytics to predict the wellness status of the players by assessing the external and internal workloads performed in previous days by using a holistic point of view. This model will be useful for sports field experts to simulate the players’ response to scheduled training in order to create a training program that maximises the training effect. Moreover, the framework developed in this study will provide insights about the prediction highlighting the external workloads features that, in a specific period of the season, affect the players’ wellness and recovery status.

## 2 Material and Methods

A framework of big data analytics was developed in this study with the aim of predicting the players’ WI based on their historical external and internal workloads and wellness status. In this section, we deeply describe the methodology used for this aim. In particular, in order to reduce any possible misleading results, this framework was developed based on the narrative review of Rossi et al. (2022) that deeply describes the correct approach to apply machine learning in sports. First of all, the GPS and wellness index data used in this study and the data pre-processing process were described in Sections 2.2, 2.3, respectively. The machine learning approach (i.e., dataset creation, models training and test, performance evaluation, and model interpretation) was provided in Section 2.4.

### 2.1 Participants

We use data of 17 players (age = 23.35 ± 5.63 years; height = 182.17 ± 6.40 cm; weight = 80.91 ± 8.34 kg) competing in Italian championship (Serie A), collected by the soccer club throughout season 2016/2017 and shared with the researchers involved in this study through a Non-Disclosure Agreement. Actually, the owner of the data is the elite soccer club that wants to remain anonymous. The club has the right to choose which information, results and data can be made publicly available and has granted access to these data to the authors of this paper only for research purposes.

### 2.2 Data

Players’ Global Position System (GPS, Viper Units 10Hz, STATSports, Newry, Ireland) data was recorded during each training and match by the club. This data obtained from this GPS was validated by previously studies (Beato et al., 2018; Bataller-Cervero et al., 2019; Beato and de Keijzer, 2019). Besides matches and training sessions, the GPS data of the national and international competitions was also recorded. In total, 2728 sessions were recorded during the season resulting in 160.47 ± 34.54 sessions per player (Supplementary Figure S1).

We extract 67 features from the GPS devices permitting to describe different aspects of the training workloads. In particular, Cinematic, Metabolic and Mechanical features quantify a player’s overall movement, the energy expenditure, and a player’s overall muscular-scheletrical load, respectively. Table 1 shows a summary of all the features extracted from GPS. Supplementary Table S1 describes in detail all the GPS features used in this study.

**TABLE 1:** Features group. Summary of all the features extracted from the GPS devices.

Feature group	List of features
Cinematic	Session time and distance; Total loading; Sprint; High speed running distance; Explosive distance; Max speed; High metabolic load; Distance covered at different velocity; Bursts duration and number, Time and distance covered above 20W; Average Estimated Metabolic Power; Equivalent Estimated Metabolic Distance
Metabolic	Time in heart zone (from 1 to 6); Max heart rate; Average Heart rate; Energy Expenditure expressed in KCal; Heart Rate Exertion
Mechanical	Impacts, Deceleration and acceleration at different intensity

About 30 min after the end of each training session or match, the players provided the Rate of Perceived Exertion (RPE). We use the CR-10 Borg’ scale, where 0 refers to the resting condition and 10 is the maximal effort that the players have ever perceived. Finally, before all of the training and match sessions, the players filled in a questionnaire about their perceived wellness (WELQUE) (Hooper and Mackinnon, 1995; McLean et al., 2010). The questionnaire consists of a 7-point Likert scale for 4 items (fatigue, sleep, stress, and muscle soreness), where 1 and 7 indicate the highest and lowest values of wellness for each item, respectively. The sum of all the items provide the overall wellness index (WI). The higher the WI is, the lower the individuals’ perceived wellness is.

### 2.3 Data Pre-Processing

All the GPS data and RPE data were normalised between 0 (i.e., minimum workload) and 1 (i.e., maximum workload) by players to reduce any intra-individual differences. Moreover, to take into consideration the history of the players, we compute Acute (moving average of the previous 7 days) and Chronic workloads (moving average of the previous 28 days) for each feature, using the exponential weighted moving average (EWMA) function to compute the moving average. EWMA is a type of rolling mean that permits to places a greater weight and significance on the most recent data as shown in Eq. 1 where *α* refers to the specify decay (see Eq. 2), *y*_*t*_ is the value at a time period *t* and *s*_*t*_ refers to the value of the EWMA at any time period *t*. The mean of *s*_*t*_ provides the EWMA workload in accordance with the time span selected (*n* = 7 and *n* = 28 for Acute and Chronic workload, respectively).

$$s_{t}=\{\begin{array}{cc} \;\;\;\;\;\;\;\;\;\;\;\;\;\;\;\;\;\;\;\;\;\;\;\;\;\;\;\;\;\;\;\;\;\;\;\;\;\;\;\;y_{t}, & if\;t=1\\ \alpha_{t}\;*\;y_{t}+\left(1-\alpha_{t}\right)\;*\;s_{t-1}, & if\;t>1 \end{array}$$ (1)

$$\alpha_{t}=\frac{2}{t+1}$$ (2)

$$workload_{EWMA}=\frac{1}{n}\overset{n}{\underset{t=1}{\sum }}s_{t}$$ (3)

Finally, we compute the ratio between Acute and Chronic workloads (ACWR) for each feature to monitor the training workload. As a matter of fact, ACWR values lower than 1 refers to training sessions where a player performs in acute less workload than “usual,” while vice versa for ACWR values higher than 1.

Table 2 provides a summary of all the pre-processing approaches used to create the dataset of this study. In total 272 features were used as predictor information in this study.

**TABLE 2:** Data pre-processing approaches description.

Data pre-processing	Description
Daily workload	Raw data of the current training/match session (67 GPS features + RPE)
Acute workload	EWMA of the previous 7 days (67 GPS features + RPE)
Chronic workload	EWMA of the previous 28 days (67 GPS features + RPE)
ACWR workload	Ration between (67 GPS features + RPE)

### 2.4 Exploiting Machine Learning Models

In this section, we describe the dataset creation (Section 2.4.1) and the two approaches used to validate the machine learning models, i.e., cross-validation and real scenario approaches. The cross-validation approach randomly splits the dataset in train and test sets (Section 2.4.2), while the real scenario approach continuously creates train and test as the season goes by (Section 2.4.3). The latter approach permits simulating what should happen if a soccer club starts using our algorithm at the beginning of the season. Moreover, in Sections Section 2.4.4, Section 2.4.5 we provide the description of the models trained on this study and the parameters used to assess the models’ prediction goodness, respectively. Finally, we provide a description of the approach used to explain the models’ decision-making process (Section 2.4.6).

#### 2.4.1 Dataset Creation

We construct a training dataset *T* consisting of a set of features *S* (272 variables) and 2728 individual training/match sessions. For each individual session *i*, we create a feature vector m_*i*_ = {*S*_*1*_ *, … ,S*_*k*_ *}* where k is the number of features that we associated with a label *c*_*i*_ referring to the WI recorded in the next day. Moreover, every feature vector composes a matrix *F*_*s*_ *=* (m_*1*_ *, … ,m*_*n-1*_) where *n* is the number of individual sessions*. F*_*s*_ is hence associated with a list of labels *C* = (*c*_*2*_ *, … ,c*_*n*_). The dataset was finally created as *T*_*s*_ = (*F*_*s*_ *,C*).

#### 2.4.2 Cross-Validation

**FIGURE 1:**
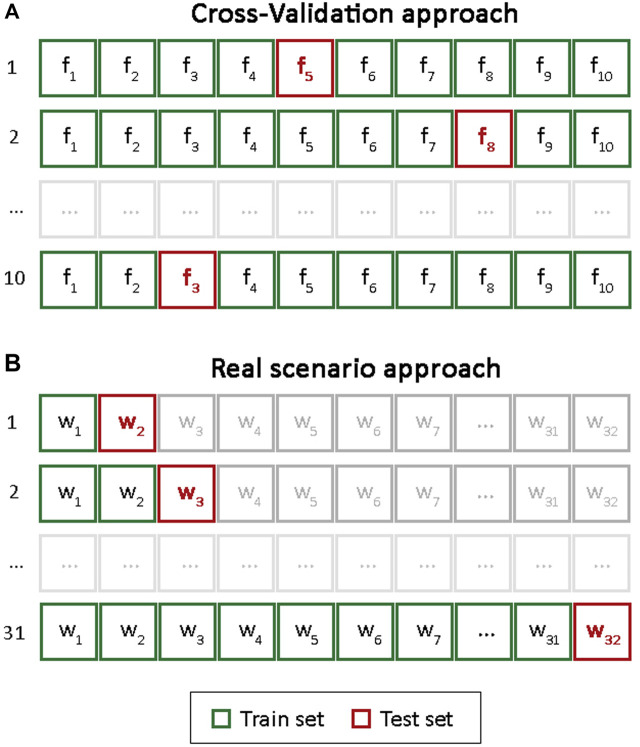
Model validation approaches. *f* and *w* refer to fold and week, respectively. **(A)** Cross-Validation approach. **(B)** Real scenario approach.

We perform 10-folds cross-validation approach to train and test the machine learning models (Figure 1A). The train and test split in each fold are performed by using a stratified approach, which permits to split the example in the dataset in the train and test sets in accordance with the distribution of WI values. In each train set, a recursive feature elimination with 3-folds cross-validation (RFECV) was performed to select the most important features to WI prediction. This approach permits to reduce the feature dimension space increasing the interpretability of the models and their accuracy. Finally, the trained models were tested in the respective test set.

#### 2.4.3 Real Scenario

Let assume that a soccer team starts recording the GPS, RPE and WI data the first day of the soccer season and it wants to develop a model that permits it to predict the WI in the next day. To this aim, we train the models on week *i* and they were tested in week *i+1*. At the end of week *i+1*, the models were re-trained using data by week *i+1* and they were tested on week *i+2*. As shown in Figure 1B, this approach was repeated by the end of the soccer season. Moreover, recursive feature elimination with 3-fold cross-validation (RFECV) was performed in each training set in order to select the best features to predict WI that permits to detect which are the workload characteristics that affects the players’ wellness by a specific week.

#### 2.4.4 Models

We train supervised machine learning models to detect patterns in the input data (GPS and RPE features) that permits to discriminate between WI classes (i.e., high, moderate, low WIs). In particular, Decision Tree classifier (DTC) and XGBoost classifier (XGB) are the two machine learning models trained in this study. RFECV allows us to extract the features importance of the fitted models expressed in percentage. Finally, to assess the validity of the models trained, we compare the prediction results with a stratified dummy classifier model (B_s_). B_s_ predict the WI classes based on WI classes’ distribution in the train set. This classifier is useful as a simple baseline to compare with the real classifiers.

#### 2.4.5 Models Performance Evaluation

Precision, Recall and F1-score for each class and the accuracy were computed to detect the model’s goodness. Precision (specificity) is the ratio of correctly predicted positive observations to the total predicted positive observations, while recall (sensitivity) is the ratio of correctly predicted positive observations to all observations in the actual class. Additionally, F1-score is the weighted mean of precision and recall. Finally, Accuracy is the ratio of correctly predicted observations to the total observations.

The model’s goodness of cross-validation approach is reported as the mean and standard deviation of performance in all of the folds, while it is the cumulative performance in the real scenario. For example, if we are testing the models in w_15_, the models’ goodness refers to all the 15 predicted weeks. This approach allows us to detect the model goodness as the season goes by.

#### 2.4.6 Models Explanation

To globally and locally explain the decision-making process of the models, we compute SHapley Additive exPlanations (SHAP) (Lundberg and Lee, 2017) values that allow us to explore the relationships between variables for predicted cases. In particular, SHAP assigns to each feature an importance value for a particular prediction (based on a linear function) permitting to evaluate the influence of each feature to final prediction by following specific rules: 1) the explanation model has to at least match the output of original model (local accuracy); 2) features missing in the original input must have no impact (missingness); 3) if we revise a model such that it depends more on a certain feature, then the importance of that feature should not decrease (consistency). Moreover, the collective SHAP values can show how much each predictor contributes, either positively or negatively, to the target variable. Understanding why a model makes a certain prediction can be as crucial as the prediction’s accuracy in many applications. Actually, inspecting the reasoning underlying the model’s decisions can provide more profound insights into the differences in WI classes.

## 3 Results

### 3.1 WI Class Distribution

**FIGURE 2:**
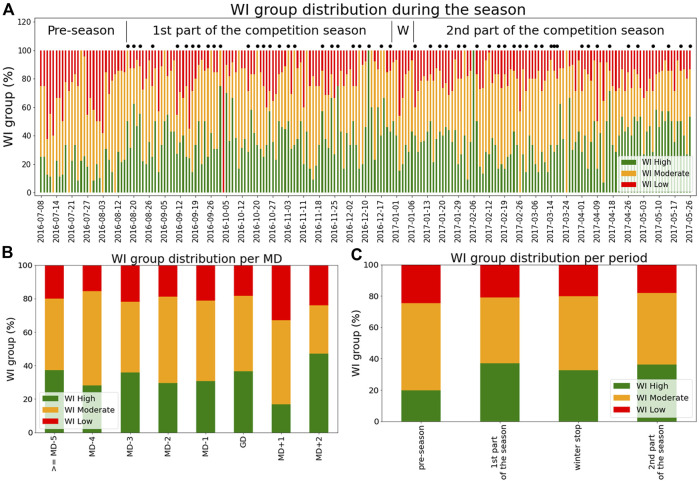
**(A)** Distribution of WI day by day as the season went by. The black dots refer to the Game Day (GD). Moreover, W refers to winter stop. **(B)** Distribution of WI in accordance with the Match Day (MD). The values refer to the day when the WI are recorded. For example, the WIs reported in Game Day (GD) refer to the WI recorded before the start of the match. **(C)** Distribution of WI in accordance with the periods of the season.

Supplementary Figure S2 and Table 3 show the distribution of WI grouped in three main classes: high WI (lower than 33rd percentile); moderate WI (between 33rd and 66th percentiles); low WI (higher than 66th percentile). Moreover, Figure 2A shows the distribution of the WI group day by day as the season goes by, while Figure 2B shows the distribution of the WI groups in accordance with the distance to the match (MD). Chi-squared test of independence shows a statistical significant frequency distribution among WI recorded in different MDs (X^2^_(df=16)_ = 42.73, *p*-value < 0.001. See Figure 2B). In particular, WIs recorded in MD+1 show higher and lower percentages of Low and High WI compared to other MDs, respectively. Additionally, we detect a high percentage of High WI in MD+2 compared to WI recorded in MD+1, MD-1, MD-2 and MD-4 (Figure 2B). Similar WI distributions were detected for all of the other MDs comparisons. Finally, WI recorded in different periods of the season results in a different distribution (X^2^_(df=6)_ = 14.31, *p*-value = 0.02, see Figure 2C). In particular, a lower number of High WI and a higher number of Moderate WI and Low HI were recorded in the Pre-season period compared to the other season periods that show a similar WI distribution.

**TABLE 3:** WI group descriptive statistics.

WI Group	Count	Mean	SD	min	25%	50%	75%	max
High	933	7.10	1.28	4	7	8	8	8
Moderate	1245	11.21	1.16	9	10	12	12	12
Low	552	15.07	1.18	13	14	16	16	17

SD, min and max refer to standard deviations, minimum values and maximal values, respectively.

### 3.2 Cross-Validation

**FIGURE 3:**
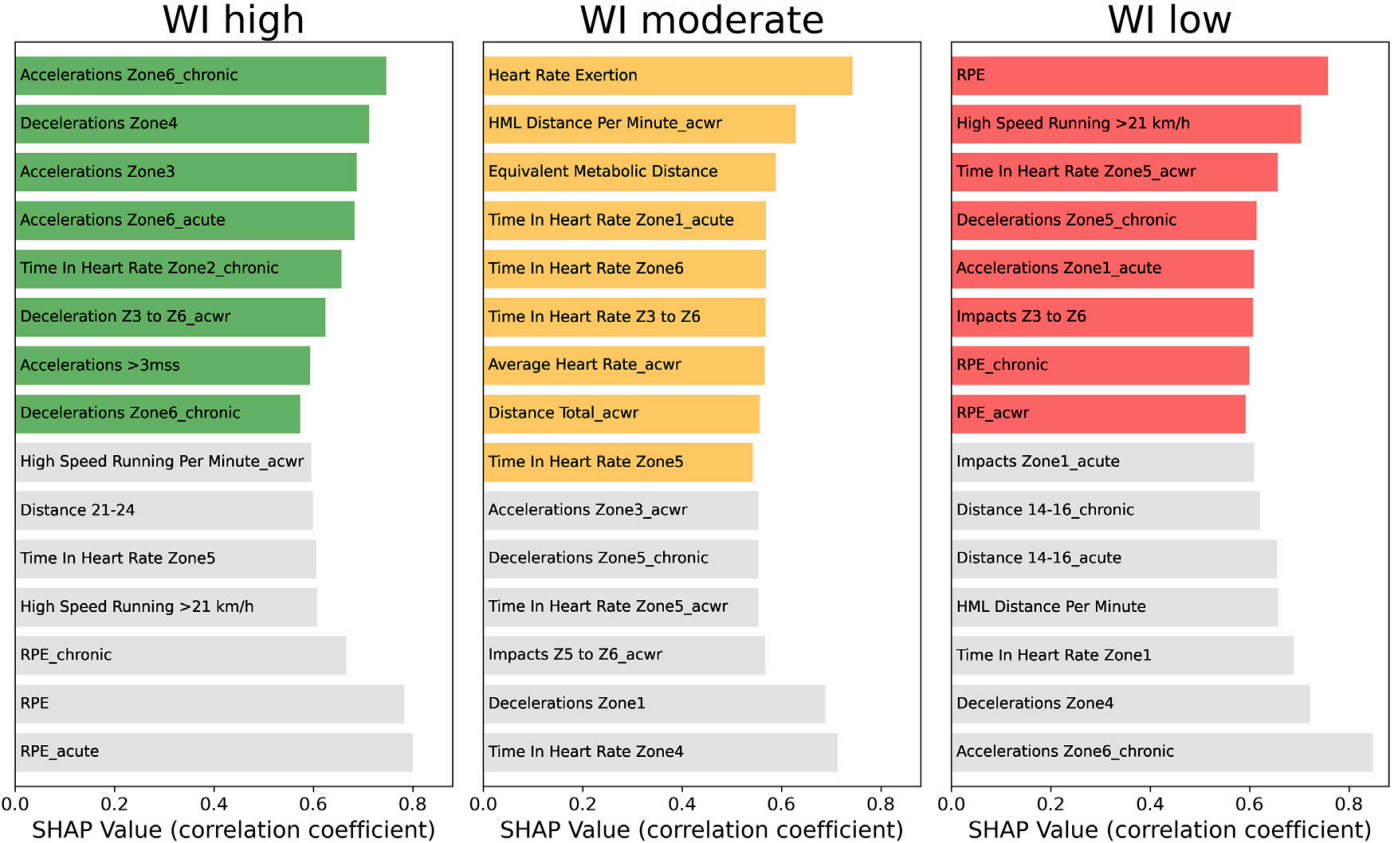
Influence of the 15 most important features of each WI class on defining classes’ membership. This plot shows the correlation coefficient between SHAP values and features’ values. Coloured bars refer to a positive correlation, while the grey ones show a negative relationship.

Table 4 shows that XGB has the higher performance (accuracy = 0.74 ± 0.01) compared with both DTC (accuracy = 0.67 ± 0.01) and B_s_ (accuracy = 0.37 ± 0.01). The low standard deviation in all the goodness parameters indicates that the models are stable and reliable. Table 5 shows the 15 most important features. 10 features out of 15 are computed as Chronic values, while only 4 and 1 features show Acute and Daily aggregations, respectively. Figure 3 shows the influence of a single variable on appertaining in a specific WI class. In particular, coloured bars show a positive influence, i.e., the higher the feature value is the higher is the probability to be in a specific WI class, while vice versa for grey bars. Supplementary Figure S3 reports the MDs’ training workloads identity card. In this figure, it is possible to assess the mean of the workload intensity performed in each MD.

**TABLE 4:** Models goodness of cross-validation.

Model	WI classes	Precision	Recall	F1-score	Accuracy
DTC	High	0.66 ± 0.01	0.67 ± 0.01	0.67 ± 0.01	0.67 ± 0.01
	Moderate	0.67 ± 0.02	0.68 ± 0.02	0.68 ± 0.02	
	Low	0.67 ± 0.02	0.68 ± 0.03	0.67 ± 0.02	
XGB	High	0.75 ± 0.01	0.75 ± 0.02	0.75 ± 0.02	0.74 ± 0.01
	Moderate	0.74 ± 0.02	0.75 ± 0.02	0.75 ± 0.01	
	Low	0.75 ± 0.03	0.73 ± 0.04	0.74 ± 0.02	
B_s_	High	0.35 ± 0.02	0.35 ± 0.03	0.35 ± 0.02	0.37 ± 0.01
	Moderate	0.38 ± 0.05	0.38 ± 0.05	0.38 ± 0.05	
	Low	0.34 ± 0.08	0.34 ± 0.09	0.34 ± 0.08	

**TABLE 5:** Feature importance of cross-validation.

Features	Folds (n)	Mean (%)	SD (%)
HML Distance Per Minute (Chronic)	10	3.73	2.15
Time In Heart Rate Zone6 (Daily)	5	3.68	3.93
Distance Total (Chronic)	4	2.66	2.86
Accelerations Zone5 (Chronic)	10	2.64	1.71
Decelerations Zone6 (Chronic)	10	2.42	1.87
Accelerations Z5 to Z6 (Chronic)	9	2.18	1.70
Impacts Zone2 (Acute)	9	2.18	0.63
Distance 16-21 (Chronic)	10	2.10	1.58
Impacts Z5 to Z6 (Chronic)	10	1.92	1.35
Energy Expenditure (KCal) (Chronic)	9	1.54	0.84
High Speed Running >21 km/h (Chronic)	10	1.53	1.33
Time In Heart Rate Zone5 (Acute)	10	1.52	1.59
Impacts Zone3 (Acute)	8	1.49	1.27
Time In Heart Rate Zone6 (Acute)	10	1.46	1.82
Distance 0-10 (Chronic)	9	1.42	1.74

This table reports only the 15 most important features. The values for mean and standard deviation (SD) are expressed in percentage. The folds number refers to how many folds a feature is used to WI prediction.

### 3.3 Real Scenario

**FIGURE 4:**
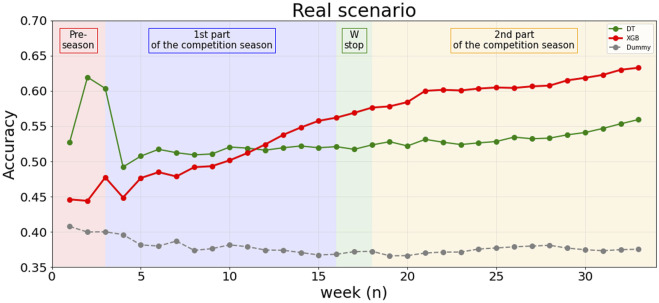
Cumulative goodness accuracy. This plot is split into four different soccer season periods: Pre-Season, 1st part of the competition season, Winter stop (W-stop) and 2nd part of the competition season.

**FIGURE 5:**
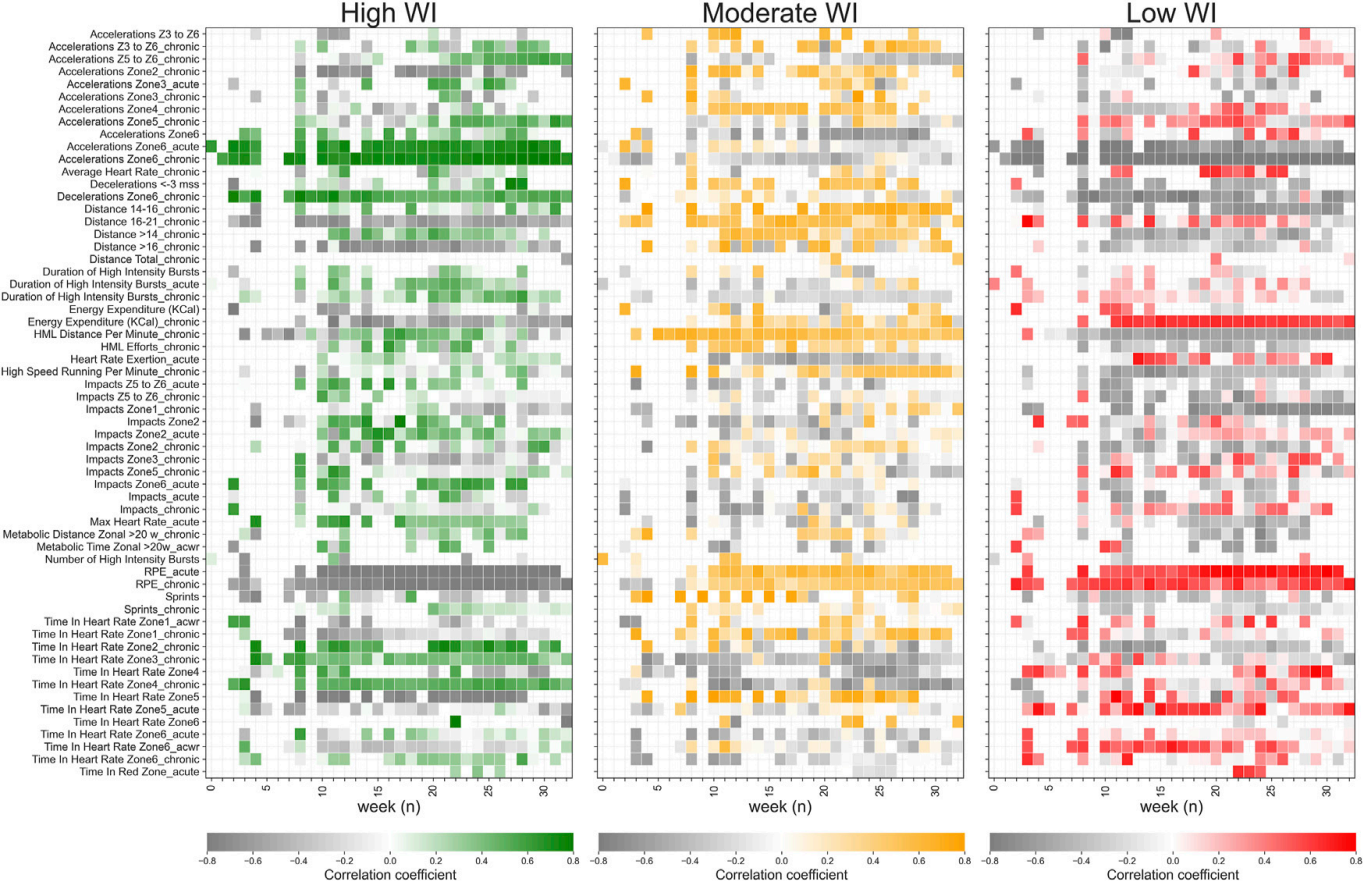
Influences of a single variable on belonging to a specific WI class. The values provided show an importance higher than 3%. Coloured bars show a positive influence, i.e., the higher the feature value is the higher is the probability to be in a specific WI class, while vice versa for grey bars.

At the end of the season, XGB shows the higher cumulative performance goodness (accuracy = 0.63) compared to DTC (accuracy = 0.56) and B_s_ (accuracy = 0.37) as shown in Figure 4. We find that XGB’s accuracy increases as the weeks go by. Actually, in the last week, XGB reached 87% accuracy (Table 6). Figure 5 shows the influence of a single variable on appertaining in a specific WI class as the season went by. This figure permits us to evaluate the change of the external and internal workloads’ influence on wellness perception. The summary of the 15 most important features in the real scenario are provided in Table 7. Almost all of the 15 most important features are Chronic (10 out of the 15 most important features in real scenario), while only 1, 1 and 2 refers to Daily, ACWR and Acute aggregated features.

**TABLE 6:** Model performance goodness of the last week.

Model	WI classes	Precision	Recall	F1-score	Accuracy
DTC	High	0.72	0.72	0.72	0.78
	Moderate	0.83	0.75	0.79	
	Low	0.78	1.00	0.88	
XGB	High	0.75	1.00	0.86	0.87
	Moderate	1.00	0.75	0.86	
	Low	1.00	0.86	0.92	
B_s_	High	0.46	0.33	0.39	0.40
	Moderate	0.50	0.55	0.52	
	Low	0.10	0.14	0.12	

**TABLE 7:** Feature importance of real scenario.

Features	Week (n)	Mean (%)	SD (%)
HML Distance Per Minute (Chronic)	23	7.66	9.64
RPE (Chronic)	8	6.65	3.97
Accelerations Zone6 (Chronic)	8	4.55	1.97
Time In Heart Rate Zone4 (Chronic)	8	3.73	1.25
Time In Heart Rate Zone3 (Chronic)	7	10.00	8.86
Decelerations Zone6 (Chronic)	6	8.08	4.72
Distance 16-21 (Chronic)	6	5.88	4.26
Impacts Zone2 (Acute)	6	3.13	0.29
Time In Heart Rate Zone5 (Acute)	5	8.47	9.16
Sprints (Daily)	5	7.59	4.92
Time In Heart Rate Zone6 (ACWR)	5	5.26	3.56
Accelerations Z5 to Z6 (Chronic)	4	5.61	2.00
Accelerations Zone5 (Chronic)	4	5.28	4.18
Accelerations Zone4 (Chronic)	4	3.49	0.58
Time In Heart Rate Zone4 (Daily)	3	28.21	24.04

This table reports only the 15 most important features. The values for mean and standard deviation (SD) are expressed in percentage. The weeks’ number refers to how many weeks a feature is used for WI prediction. The values are sorted by week and mean.

## 4 Discussion

This study provides a framework of big data analytics that investigates the relationship between training workloads and players’ wellness status of the next day. Actually, the main findings of this study is that it is possible to accurately estimate WI of the players based on their workload history and in particular by the chronic feature aggregation (exponential weighted average of the training workloads of the previous 28 days). This model may help athletic trainers and coaches to better schedule training in order to enhance the training adaptations based on the match periods and consequently to the distance from the match day (weekly microcycle).

The period of the season and distance to the match day (MDs) are two factors that affect the distribution of WI classes (Figure 2). In particular, Figure 2C shows that the pre-season period (i.e., general preparation phase) results in lower wellness status compared to the other periods of the season (i.e., in-season and winter stop). Actually, the perceived wellness has been found to be related to physiological demands that may vary according to training methods and workloads schedules (Fessi et al., 2016; Govus et al., 2017). As a matter of fact, during the pre-season period, the training sessions are usually scheduled in order to reestablish the soccer players’ fitness after the summer stop (Bangsbo et al., 2006; Jeong et al., 2011) resulting in higher training workloads, i.e., the frequency and mean duration of each training session were found to be significantly higher compared to the competitive period (Bangsbo et al., 2006; Jeong et al., 2011). Differently, the in-season period is focused to enhance/maintain physical capacities and develop techno-tactical skills according to players’ positional roles showing lower training workloads and higher players’ wellness status compared to pre-season period (Bangsbo et al., 2006; Jeong et al., 2011). Moreover, during the competitive period (in-season), the workloads are scheduled in accordance with the weekly microcycles (Rossi et al., 2016) resulting in a different distribution of WI classes and training workloads on each match day (Figure 2B). Actually, the day after the match (MD+1) shows the lowest wellness status that may be induced by the high workloads performed by the soccer players during the match. Differently, in MD+2, the players showed the highest wellness status (Figure 2B) because the weekly day-off (MD+1) had permitted a complete recovery from the effort performed during the MD (Gallo et al., 2017). Knowing when altered wellness status returns to the high class (High WI) may lead athletic trainers and coaches to prescribe the heaviest load during the week in accordance with the distance from and to the match day (Gallo et al., 2017).

To the best of our knowledge, this is one of the first studies investigating the multidimensional relationship between training workloads and wellness status throughout an entire soccer season. In particular, previous studies focused on predicting the players’ wellness status by a multidimensional approach investigated only the pre-season period highlighting that self-reported wellness combined with GPS technology may enhance the understanding of training responses and inform program development (Fields et al., 2021). Moreover, monodimensional approach’s studies demonstrated a strong correlation between the training load (TL, i.e., monodimansional variable computed as the product between the duration of the training session and the rate of perceived exertion) and the wellness status (Clemente et al., 2019; Op De Beéck et al., 2019; Clemente et al., 2020). For example, Perri et al. (2021) highlight the fact that WI is predictable by TL with an accuracy of about 41%. Actually, the multidimensional approach proposed in this study shows a more accurate prediction ability to detect the wellness status of the soccer players compared to the monodimensional one. In particular, the XGB model shows an accuracy of about 74% demonstrating that the GPS workloads data help to better (Table 4) understand the relationship between external load and wellness status instead of using only the TL parameter. Moreover, the prediction ability of the algorithm increases as the season goes by reaching an accuracy of about 87% in the last week of the soccer season (Table 6) resulting in a cumulative accuracy of 63% (Figure 4). The higher the number of the examples for training the algorithm is, the higher is the prediction ability of the machine learning algorithm. As a matter of fact, XGB cumulative accuracy does not reach a steady-state phase during the 34 weeks of soccer season but it continuously increases as the season goes by.

The no perfect prediction ability of XGB could be explained by the fact that not only external workloads (i.e., metabolic, cinematic, and mechanical workload features) affect the wellness status, but also psychological factors, contextual features, and recovery-oriented activities (e.g., improved diet, cold-water immersion, stretching, and sleep) could have an impact on players’ wellness (Rossi et al., 2017; Rossi et al., 2019; Perri et al., 2021). Future works are scheduled in order to solve this gap. However, in this study, we evaluate only the effect of the external (GPS features) and internal (RPE) training workloads on perceived wellness status of the soccer players. In particular, Tables 5, 7 show the most important features to predict WI in cross-validation and evolutive scenarios, respectively. Actually, Figure 3 shows a general overview of the influence of each feature on each WI class for this soccer club during the entire soccer season. This plot provides insight about which and how external and internal workload features affect the players’ wellness status. In particular, colored bars refer to features that positive induce a players to be part at one of the WI classes (the higher these features are the higher the probability to be part of a WI class is), while grey bars show a negative influence (the higher these features are the lower the probability to be part of a WI class is). To be noticed that these features are relevant only for the soccer team analysed in this study. However, future works are needed in order to assess if these predictive models are generalizable/transferrable to other soccer teams or different seasons of the same soccer team. Actually, the players’ wellness status could be affected by, for example, the training schedule, the individual’s characteristics, and coaching style that may vary the players’ response to internal and external training workloads. As a matter of fact, the features’ importance and consequently the models’ rules for WI classification changes in accordance with the period of the soccer season where the physiological demands, individuals’ physical status, and players’ readiness are different (Figure 5).

Chronic workloads features show the highest importance for predicting WI (cross-validation = 0.98 ± 0.63%; evolutive scenario = 1.20 ± 1.08%) followed by acute (cross-validation = 0.53 ± 0.33%; evolutive scenario = 0.74 ± 0.56%), daily (cross-validation = 0.50 ± 0.43%; evolutive scenario = 0.65 ± 0.59%) and acwr (cross-validation = 0.437 ± 0.14%; evolutive scenario = 0.42 ± 0.29%) ones. The strong relationship between chronic and acute workloads was already detected in a few previous studies (McLaren et al., 2018; Clemente et al., 2019; Op De Beéck et al., 2019; Clemente et al., 2020; Nobari et al., 2021), which highlighted the fact that this relationship changes in accordance with the periods of the soccer season. This result is corroborate also in our study. In particular, the machine learning approach proposed in this study permits to detect the influence of each feature in each WI class week by week (Figure 5) allowing to deeply understand the external and internal workloads influence on the players’ wellness status in relationship with the period of the soccer season linked for example to the different players’ status (both physical and psychological) and match schedule. Of note, the importance of the features changes as the season goes by resulting sometime in an alternate influence (positive and negative) of the features on each WI class. For example, HML distance per minute with chronic aggregation shows a negative influence in the first part of the season (until week 11) and in the end of the soccer season (after week 26), but has a positive influence in the competitive part of the season (between week 11 and 26). Differently, other features such as Acceleration in zone 6 (both with chronic and acute aggregation), Time in heart zone 2 (chronic) and Time in heart zone 3 (chronic) show a positive influence on the players’ wellness status during the entire season, while RPE has a negative impact. Actually, the interaction among daily, acute and chronic features leads the machine learning algorithms to accurately predict WI classes.

The machine learning framework of big data analytics proposed in this study may have practical relevance for athletic trainers and coaches allowing to improve the decision-making process during scheduling the training workloads program by simulating it. In particular, the insights derived from impact of the workload features permit to assess the external and internal stimuli affecting the players’ wellness status in each period of the soccer season allowing to maximise the training effect.

## 5 Limitation of the Study

The main limitation of this work is the results shown in this study are only valid for this specific team. Different players’ characteristics, soccer level, competitive demands, and training program results in different physiological demands and consequently in different wellness response to the external and internal stimuli. Hence, in this study, we provide an analytical approach that can be developed for each team creating personalized decision-making rules that predict the players’ wellness status by external and internal workloads. Future works are needed in order to assess the different influences of training workloads of several teams with different levels of competition, different age groups, different gender, and different training programme. Moreover, the second limitation of this work is that the wellness status of the players is evaluated by a self-reported approach. Even if these metrics are widely used in practice to assess the status of the athletes, it has not undergone a rigorous evaluation of their validity and reliability. Finally, the last main limitation of this work is that we did take into consideration contextual factors (e.g., metrological status, distance to the match, and championship schedule) and individual characteristics (e.g., injury risk and fit status) that could affect the influence of training workloads to wellbeing status of the athletes. Future works need to provide more and more details about the contextual factors and individuals’ characteristics in order to have a more holistic point of view of players’ status.

## 6 Conclusion

The strong correlation detected between WI and training workloads permits to detect patterns affecting the wellness status of the soccer players. Consequently, the machine learning algorithms proposed in this study may be useful for sport scientists, athletic trainers, and coaches to maximise the periodization of the training based on the physiological requests of a specific period of the season. Hence, by using this machine learning framework, field experts should have a complete overview of the individual mechanisms that influence changes in players’ perceived wellness.

## Data Availability

The datasets presented in this article are not readily available because The soccer club shared the data with the researchers involved in this study through a Non-Disclosure Agreement. Actually, the owner of the data is the elite soccer club that wants to remain anonymous. The club has the right to choose which information, results and data can be made publicly available and has granted access to these data to the authors of this paper only for research purposes. Requests to access the datasets should be directed to Alessio Rossi, alessio.rossi2@gmail.com.
