# Supplementary material for: Wellness Forecasting by External and Internal Workloads in Elite Soccer Players: A Machine Learning Approach
Source: Front Physiol. 2022 Jun 15;13:896928. doi: 10.3389/fphys.2022.896928 (PMC9240643; doi:10.3389/fphys.2022.896928)
Supplement: Supplementary file 1 [file Table1.DOCX]

Supplementary Material

**Table S1.** GPS feature description. In this table is reported only the main GPS features selected by the machine learning models for predicting WI.

| **Category** | **Feature** | **Description** |
| --- | --- | --- |
| Cinematic | Distance Total | Distance in meters covered during the training session |
|  | Distance 14-16 | Distance in meters covered between 14 and 16 km/h |
|  | Distance 16-21 | Distance in meters covered between 16 and 21 km/h |
|  | Distance >14 | Distance in meters covered above 14 km/h |
|  | Distance >16 | Distance in meters covered above 16 km/h |
|  | HML Distance Per Minute | Distance in meters per minute covered with a Metabolic Power above 25.5W/Kg |
|  | HML Efforts | Summed numbers of accelerations, decelerations, high speed running |
|  | High Speed Running Per Minute | Distance in meters per minute covered above 5.5m/s |
|  | Metabolic Distance Zonal >20 w | Distance in meters covered above 20 watt per Kg |
|  | Metabolic Time Zonal >20w | Time in seconds covered above 20 watt per Kg |
|  | Number of High Intensity Bursts | Number of events with acceleration ≥ 4.0 m/s^2^, deceleration ≤ −4.0 m/s^2^, or impacts ≥ 11 G |
|  | Duration of High Intensity Bursts | Time in second spent with acceleration ≥ 4.0 m/s^2^, deceleration ≤ −4.0 m/s^2^, or impacts ≥ 11 G |
| Metabolic | Average Heart Rate | Mean of the Heart Rate during the training session |
|  | Max Heart Rate | Maximal Heart Rate during the training session |
|  | Heart Rate Exertion | Time spent in each heart rate zone multiplied by a  weighing factor |
|  | Time In Heart Rate Zone1 | Time in seconds spent at <55% of the HR max |
|  | Time In Heart Rate Zone2 | Time in seconds spent at 55-65% of the HR max |
|  | Time In Heart Rate Zone3 | Time in seconds spent at 65-75% of the HR max |
|  | Time In Heart Rate Zone4 | Time in seconds spent at 75-80% of the HR max |
|  | Time In Heart Rate Zone5 | Time in seconds spent at 80-85% of the HR max |
|  | Time In Heart Rate Zone6 | Time in seconds spent at 85-90% of the HR max |
|  | Time In Red Zone | Time in seconds spent at >90% of the HR max |
|  | Energy Expenditure (KCal) | Amount of energy spent by a player during training |
| Mechanic | Accelerations Zone2 | Number of accelerations between 1.7 and 3.3 m/s^2^ |
|  | Accelerations Zone3 | Number of accelerations between 3.4 and 3.9 m/s^2^ |
|  | Accelerations Zone4 | Number of accelerations between 4.0 and 5.0 m/s^2^ |
|  | Accelerations Zone5 | Number of accelerations between 5.0 and 6.3 m/s^2^ |
|  | Accelerations Zone6 | Number of accelerations above 6.4 m/s^2^ |
|  | Accelerations Z3 to Z6 | Number of accelerations above 3.4 m/s^2^ |
|  | Accelerations Z5 to Z6 | Number of accelerations above 5.0 m/s^2^ |
|  | Decelerations <-3 m/s^2^ | Number of decelerations below 3 m/s^2^ |
|  | Decelerations Zone6 | Number of decelerations above 6.4 m/s^2^ |
|  | Impacts | Number of events with magnitude >2.0 G |
|  | Impacts Zone1 | Number of events with magnitude <6.0 G |
|  | Impacts Zone2 | Number of events with magnitude 6.1-6.5 G |
|  | Impacts Zone3 | Number of events with magnitude 6.6-7.0 G |
|  | Impacts Zone4 | Number of events with magnitude 7.1-8.0 G |
|  | Impacts Zone5 | Number of events with magnitude 8.1-10.0 G |
|  | Impacts Zone6 | Number of events with magnitude >10.1 G |
|  | Impacts Z5 to Z6 | Number of events with magnitude >8.1 G |
|  | Sprints | Number of events over 7.0 m/s (25.2 km/h) |


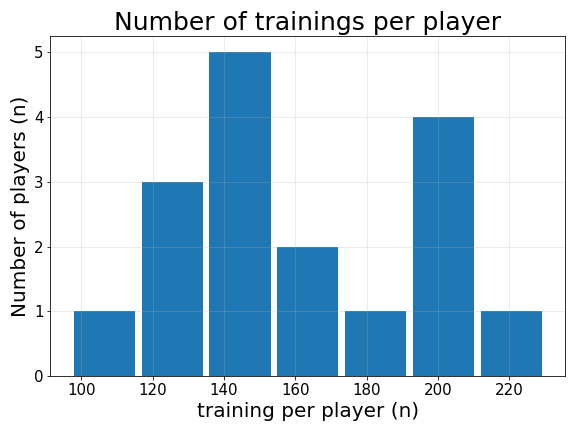


**Figure S1.** The number of sessions per player recorded.


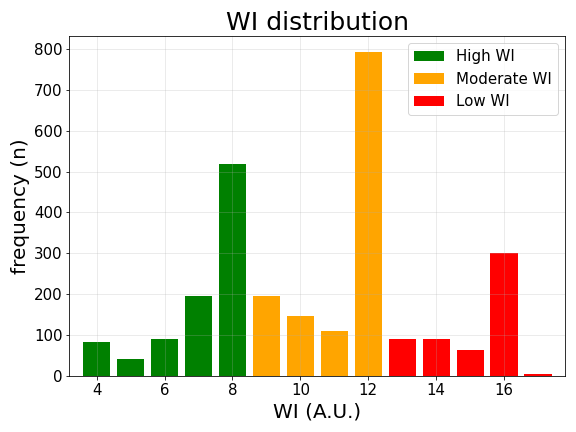


**Figure S2.** WI distribution grouped into three main classes: i) high WI; ii) moderate WI; iii) low WI.

**
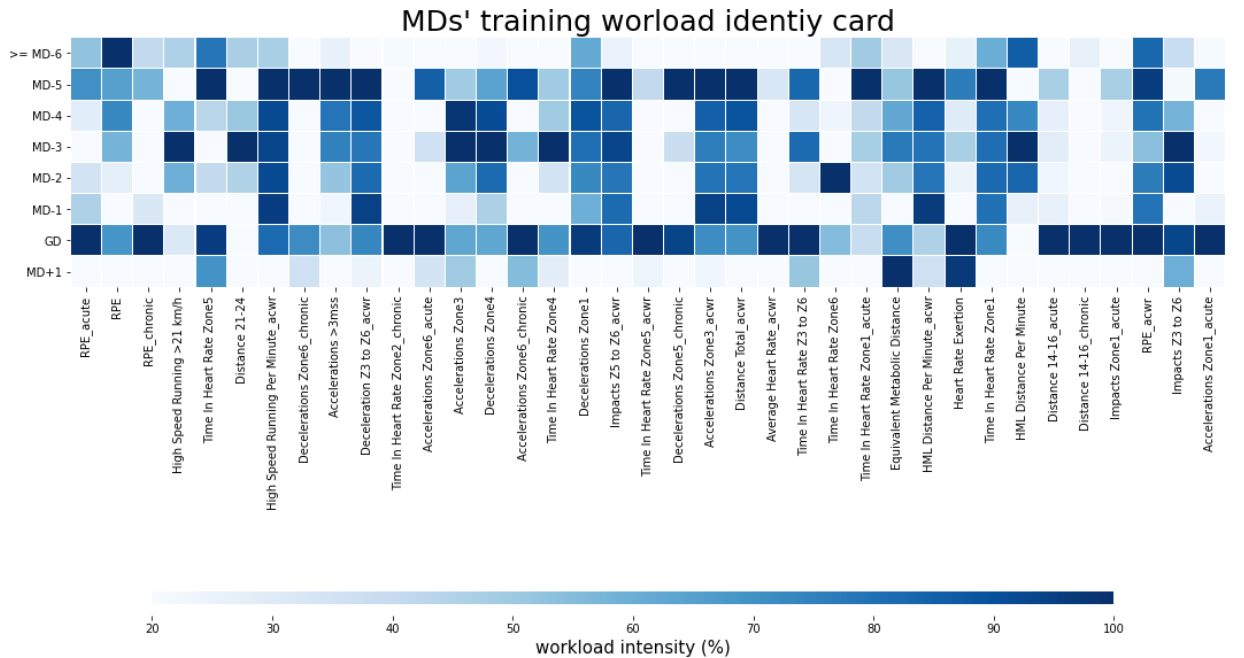
**

**Figure S3.** Team’s identity card (heatmap) of the training workloads performed in each match day (MD). The darker the colour is, the higher the workload is. The workload values reported in this figure reflects the mean of the normalized values for each feature in each MD during the entire soccer season.
